# Early Risk Factors for Retinopathy of Prematurity in Very and Extremely Preterm Chinese Neonates

**DOI:** 10.3389/fped.2020.553519

**Published:** 2020-10-15

**Authors:** Hai-Bo Huang, Yi-Hua Chen, Jing Wu, Matt Hicks, Yan-Zhi Yi, Qian-Shen Zhang, Chun-Bong Chow, Po-Yin Cheung

**Affiliations:** ^1^Department of Pediatrics, University of Hong Kong-Shenzhen Hospital, Shenzhen, China; ^2^Department of Ophthalmology, University of Hong Kong-Shenzhen Hospital, Shenzhen, China; ^3^Department of Pediatrics, University of Alberta, Edmonton, AB, Canada

**Keywords:** prematurity, retinopathy of prematurity, hypotension, blood gases, prediction

## Abstract

**Objective:** To investigate the incidence and risk factors of retinopathy of prematurity (ROP) in very and extremely preterm (28^+0^- <32^+0^, and <28^+0^ weeks gestation, respectively) neonates, and the predictive factors for ROP in the early hours after birth and during hospitalization.

**Methods:** Using a prospective database supplemented with a retrospective chart review, we identified preterm neonates born at gestation <32 weeks at the University of Hong Kong-Shenzhen Hospital between January 2015 and August 2018. Demographic and clinical variables were studied including indicators of disease acuity in the first 24 h after birth. We also compared the difference in risk factors between survivors with ROP and survivors without ROP.

**Results:** During the study period, there were 529 preterm neonates admitted to our neonatal intensive care unit with 120 (23%) born at <32 weeks' gestation. Thirteen (11%) neonates died. Among the 107 survivors, 23 (21%) had ROP, of whom five (22%) received laser and/or medical therapy for severe ROP. Compared with survivors without ROP, infants with ROP had lower mean blood pressure in the first 12 and 24 h after birth, respectively. Using multivariate regression, gestation age, mean blood pressure in the first 12 h after birth, hospital length of stay, and total days of blood gases pH <7.2 were independent risk factors for ROP.

**Conclusions:** In this small cohort of Chinese neonates born <32^+0^ weeks' gestation, survivors with ROP had a lower blood pressure in the early hours after birth, younger gestation, longer hospital stay, and duration of acidosis when compared to those without ROP.

## Introduction

Improved obstetrical and neonatal care has increased the survival of very preterm (28^+0^- <32^+0^ weeks gestation) and extremely preterm (<28^+0^ weeks gestation) neonates, who may subsequently develop complications including retinopathy of prematurity (ROP). Retinopathy of prematurity is a developmental, proliferative, vascular disorder that occurs in the retina of preterm infants who have incomplete retinal vascularization (1). Globally, it was estimated that 184,700 of 14.9 million preterm neonates (1.2%) developed any stage of ROP, with an incidence of 16% in the very and extremely preterm neonates (2). In high-income countries, ~20% of infants with ROP would subsequently require treatment (2). However, in China, the incidence of ROP was 6.6–28.1% with up to 40% (13.2–39.9%) with severe type I ROP requiring laser and/or medical therapy (3–6).

The most important risk factor for developing ROP is prematurity. In multivariate analyses, other independent risk factors for ROP include low birth weight (BW), low gestational age (GA), assisted ventilation for longer than 1 week, surfactant therapy, high blood transfusion volume, cumulative illness severity, low caloric intake, hyperglycemia, and insulin therapy (7–14). However, there is little information regarding predictive factors for ROP in the early hours after birth.

We aimed to investigate the incidence and risk factors of ROP in the very and extremely preterm neonates. We hypothesized that the indicators of disease acuity including blood pressure (BP) during the early hours after birth would be associated with the subsequent development of ROP.

## Methods

This study was performed using a prospective database supplemented with retrospective chart reviews. The study received the approval of institutional ethics board [#(2018)88r]. Informed consent was obtained from the parents before the initial screening for ROP as per institutional practice.

All neonates born before 32 weeks' gestation between January 2015 and August 2018 were included in this study. Infants were excluded from the study if they had congenital retinal abnormalities or if a follow-up eye examination was not completed or non-survival before initial hospitalization. All infants who were admitted into the Neonatal Intensive Care Unit of the University of Hong Kong-Shenzhen Hospital had demographic and clinical information collected in a prospective database by clinical assistants supervised by a senior doctor (Y-H C). The demographic and clinical information included GA, BW, gender, delivery mode, single or multiple births, Apgar score at 1 and 5 min, mean BP, lowest body temperature, urine output in the 1st and 2nd 12 h periods of life, vasoactive medications in the 1st 24 h, hospital length of stay, duration of total parenteral nutrition (TPN) and ventilation (non-invasive and invasive), intubation in the delivery room, surfactant therapy, weight gain, respiratory distress syndrome, bronchopulmonary dysplasia (BPD), intraventricular hemorrhage, necrotizing enterocolitis, neonatal sepsis, total number of days with blood gases pH <7.2, pH > 7.5, PCO_2_ <30 mmHg, PCO_2_ > 80 mmHg, PO_2_ <50 mmHg, PO_2_ > 150 mmHg, highest C-reactive protein level, lowest leukocyte and platelet counts, and number of red blood cells transfusion.

Eye examinations were performed under indirect ophthalmoscopy by JW, who has extensive experience in neonatal retinal examination, on all very and extremely preterm infants according to the unit policy. The International Classification of ROP guidelines were used to record the stage of ROP, location by zone, and signs of plus disease (15). All infants were scheduled to have their first examination between 4 and 6 weeks of life. If ROP was detected, the examinations were repeated at intervals according to the severity of findings. Ophthalmic examinations were continued until full retinal vascularization and the maximum stage of ROP for each infant was reported. The data were analyzed based on the most advanced stage of ROP in the eye with the most severe disease. Severe ROP was defined as ROP that required treatment for laser photocoagulation and/or intravitreal bevacizumab. Criteria for treatment of ROP were based on the Early Treatment for ROP (16).

Among demographic and clinical information in the clinical database, BPD was defined as oxygen dependency beyond 28 days after birth and required ventilatory support and/or supplemental oxygen at 36 weeks of post-menstrual age (17). Sepsis was defined by the presence of a positive blood culture with clinical symptoms and signs. In our unit, we transfused infants with 15 mL/kg of red blood cells each time under the discretion of the clinical team.

## Statistical Analysis

Statistical analyses were conducted using IBM Statistical Product and Service Solutions software Version 24 (SPSS Inc., Chicago, IL). Continuous data were examined as mean ± standard deviation and median and interquartile ranges for parametric and non-parametric variables, respectively. Comparisons of categorical variables were performed using the Fisher's exact test. Variables with *p*-value < 0.2 in the univariate analysis were entered into a backward stepwise multivariate logistic regression model to identify the association with ROP. Odds ratios (ORs) and 95% confidence intervals (CIs) were calculated. Model fitness was assessed with the Hosmer–Lemeshow goodness-of-fit test. Area under the curve (AUC) of the receiver operating curves (ROC) was analyzed to assess the prognostic performance. Sensitivity, specificity, and positive and negative predictive values were also determined. All probability values were two-sided and a *p*-value < 0.05 was considered statistically significant.

## Results

From January 2015 to August 2018, 529 preterm neonates were admitted to our NICU with 120/529 (23%) born at <32 weeks' gestation. Of these 120 neonates, there were 36 (30%) extremely preterm neonates of <28^+0^ weeks and 84 (70%) very preterm neonates of 28^+0^ to 31^+6^ weeks' gestation. Thirteen (11%) neonates died. The 107 survivors (24 extremely preterm and 83 very preterm infants) met criteria for ROP screening and were included with complete retinal follow-up examinations. Retinopathy of prematurity was diagnosed in 23 (21%) of 107 infants. Five (4.7% of 107; 22% of 23) infants had severe type I ROP that required laser and/or medical therapy. Fifteen (63%) and 3 (12.5%; 20% of 15) of 24 surviving extremely preterm infants had ROP and severe type I ROP, respectively.

The surviving infants with ROP had younger GA and lower BW than that of survivors without ROP [27.6 (26.4–28.3) vs. 30.3 (28.9–31.3) weeks and 988 ± 243 g vs. 1,383 ± 320 g, respectively, both *p* <0.001] (Table 1). In univariate analyses, risk factors for ROP were categorized into those within the first 24 h of life (early hours) and during the hospital stay. Risk factors for ROP included Apgar score at 1 and 5 min, mean BP, lowest body temperature, urine output, vasoactive medications, intubation in the delivery room, respiratory distress syndrome, and surfactant therapy in the first 24 h (Table 1). Sex, delivery mode, multiple birth, lowest body temperature, and urine output during the first 24 h after birth were not different between infants with and without ROP.

**Table 1:** Univariate analysis of risk factors for retinopathy of prematurity in the first 24 h after birth and during hospitalization (mean ± SD) [median (IQR)].

**Variables**	**ROP (*n* = 23)**	**No ROP (*n* = 84)**	***p*-value**
**FIRST 24 H AFTER BIRTH**			
Gestational age (weeks)	27.6 (26.4–28.3)	30.3 (28.9–31.3)	<0.001
Birth weight (grams)	988 ± 243	1,383 ± 320	<0.001
Male gender	13 (57%)	42 (50%)	0.642
Vaginal delivery	14 (60%)	35 (42%)	0.156
Multiple birth	10 (43%)	24 (29%)	0.209
Apgar score at 1 min	6 (5–7)	8 (6–9)	<0.001
Apgar score at 5 min	8 (7–9)	9 (8–10)	0.001
Mean BP in 0–12 h (mmHg)	35 ± 5	41 ± 5	<0.001
Mean BP in 12–24 h (mmHg)	38 ± 7	45 ± 7	<0.001
Vasoactive medications (0–24 h)	6 (26%)	4 (5%)	0.006
Lowest temperature in 0–12 h (°C)	36.1 (35.2–36.5)	36.4 (35.8–36.7)	0.108
Lowest temperature in 12–24 h (°C)	36.7 (36.5–36.9)	36.8 (36.5–37)	0.775
Urine output in 0–12 h (ml/Kg/h)	2.0 (1.3–3.5)	2.9 (2.0–3.6)	0.141
Urine output in 12–24 h (ml/Kg/h)	4.0 ± 1.5	3.7 ± 1.5	0.458
Intubation in the delivery room	11 (48%)	16 (19%)	0.013
Respiratory distress syndrome	21 (91%)	59 (70%)	0.056
Surfactant therapy	15 (65%)	19 (23%)	<0.001
**DURING HOSPITALIZATION**			
Hospital stay (days)	76 (58–109)	43 (32–60)	<0.001
Weight gain (grams/day)	20.6 ± 4.9	22.1 ± 5.5	0.242
Duration of parenteral nutrition (days)	32 (27–53)	14 (9–22)	<0.001
Duration of invasive ventilation (days)	4 (0–13)	0 (0–0)	<0.001
Duration of non-invasive ventilation (days)	42 (35–48)	10 (4–34)	<0.001
Total days of pH <7.2	1 (0–5)	0 (0–0)	<0.001
Total days of pH > 7.5	0 (0–1)	0 (0–0)	0.236
Total days of PCO_2_ <30 mmHg	1 (0–2)	0 (0–1)	0.311
Total days of PCO_2_ > 80 mmHg	0 (0–1)	0 (0–0)	0.001
Total days of PO_2_ <50 mmHg	5 (1–12)	0 (0–2)	<0.001
Total days of PO_2_ > 150 mmHg	0 (0–0)	0 (0–0)	0.031
Bronchopulmonary dysplasia	20 (87%)	25 (30%)	<0.001
Intraventricular hemorrhage	5 (22%)	9 (11%)	0.175
Necrotising enterocolitis	4 (17%)	1 (1%)	0.007
Neonatal sepsis	7 (30%)	3 (4%)	0.001
Highest C-Reactive Protein during hospital stay (mg/L)	11 (4–30)	2 (1–4)	<0.001
Number of packed red blood cell transfusions	2 (1–4)	0 (0–1)	<0.001
Lowest white blood cell count during hospital stay (ˆ 10^9^/L)	5.73 (3.96–9.05)	6.24 (5.05–8.04)	0.319
Lowest platelet count during hospital stay (ˆ 10^9^/L)	140 (40–187)	204 (165–243)	<0.001

*Intraventricular hemorrhage (Papille's stage 3 or 4) and necrotising enterocolitis (Bell's stage) were included for analysis*.

Factors during hospital stay which were different between surviving infants with and without ROP included total number of days of PO_2_ <50 mmHg, PO_2_ > 150 mmHg, PCO_2_ > 80 mmHg, blood gas pH <7.2, days of non-invasive and invasive ventilation support, hospital length of stay, duration of parenteral nutrition, diagnosis of BPD, necrotizing enterocolitis, or sepsis, highest C-reactive protein levels, lowest platelet count, and number of red blood cell transfusions (Table 1). Weight gain during the hospitalization, occurrence of intraventricular hemorrhage, and lowest leukocyte count were not different between the two groups.

Based on a backward stepwise multivariate logistic regression model to predict ROP using risk factors with *p*-value < 0.2 in univariate analyses, GA was the only statistically significant independent risk factor (*p* < 0.001) in the first 24 h of life whereas mean BP in the first 12 h was modest (*p* = 0.057) (Table 2). Other statistically significant independent risk factors included hospital stay and total days of acidosis (pH <7.2) (Table 2). Duration of non-invasive ventilation, necrotizing enterocolitis, neonatal sepsis, and blood transfusion were not statistically significant in the regression analysis of ROP (*p* = 0.061–0.087). In this model, odds ratios of Apgar scores, BP at 12–24 h, lowest temperature in 0–12 h, vasoactive medications used in 0–24 h, intubation in the delivery room, respiratory distress syndrome, surfactant therapy, duration of parenteral nutrition, total days of PCO_2_ > 80 mmHg, PO_2_ <50 mmHg and >150 mmHg, BPD, and lowest platelet count had *p*-values > 0.1 (data not shown).

**Table 2:** Independent risk factors of retinopathy of prematurity in the first 24 h after birth and during hospitalization^a^ in infants born at <32 weeks gestation.

	***B*-value**	**SE value**	**Wals value**	**OR value^b^**	**95% CI**	***p*-value**
**FIRST 24 H AFTER BIRTH**						
Gestational age	−0.889	0.224	15.725	0.411	0.265–0.638	<0.001
Mean blood pressure in 0–12 h	−0.126	0.066	3.622	0.882	0.775–1.004	0.057
**DURING HOSPITALIZATION**						
Hospital stay	0.104	0.034	9.526	1.110	1.039–1.185	0.002
Duration of non-invasive ventilation	−0.043	0.023	3.508	0.957	0.915–1.002	0.061
Total days of pH <7.2	−0.715	0.323	4.892	0.489	0.260–0.922	0.027
Necrotising enterocolitis	3.768	2.194	2.950	43.298	0.588–3190.097	0.086
Neonatal sepsis	1.971	1.082	3.319	7.176	0.861–59.800	0.068
Number of packed red blood cell transfusions	0.677	0.395	2.938	1.968	0.907–4.269	0.087

a *Variables with p <0.2 in the unadjusted analysis (Table 1) were entered into a backward stepwise multivariate logistic regression analysis*.

Data from all very and extremely preterm infants (n = 107) were analyzed to predict

b *ROP (n = 23)*.

**Figure 1:**
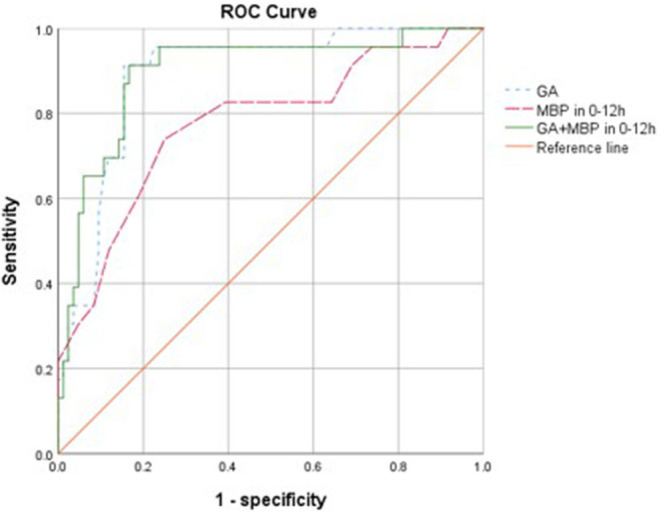
Receiver operating characteristic (ROC) curves for the ability of gestational age (GA), mean blood pressure (MBP) 0–12 h, and GA combined with MBP in 0–12 h in models to predict the development of retinopathy of prematurity with <32 weeks (*n* = 107). The reference line has an area of 0.50.

To assess the potential to predict ROP using risk factors in the first 24 h, we examined the predictive value of mean BP and GA. Mean BP in the first 12 h, GA, and their combination appeared to be significant predictors and achieved an AUC of 0.78 (95% CI 0.66–0.89, *p* < 0.001), 0.89 (95% CI 0.82–0.96, *p* < 0.001), and 0.90 (95% CI 0.82–0.97, *p* < 0.001), respectively, for predicting the development of ROP (Figure 1). The optimal cutoff values for GA and mean BP were 28.3 weeks and 35 mmHg, respectively. The sensitivity, specificity, positive and negative predictive values of GA (<28.3 weeks), and mean BP (<35 mmHg) in the first 12 h for the development of ROP are shown in Table 3. Taken together, the positive predictive value was highest with the combination of GA and mean BP (0.72), whereas a GA <28.3 weeks had the highest negative predictive value of 0.97.

**Table 3:** Predictive characteristics of gestational age (GA) and mean blood pressure (BP) in the first 12 h after birth for the development of retinopathy of prematurity.

	**Cut off value**	**TP**	**FP**	**FN**	**TN**	**Sensitivity**	**Specificity**	**PPV**	**NPV**	**Odds ratio**	**Relative risk**	**Accuracy rate**
GA (weeks)	28.3	21	2	13	71	0.91	0.85	0.62	0.97	57.35	22.54	0.86
Mean BP in 0–12 h (mmHg)	35	14	9	16	68	0.61	0.81	0.47	0.88	6.61	3.99	0.77
Mean BP in 0–12 h combined with GA		13	10	5	79	0.57	0.94	0.72	0.89	20.54	6.43	0.86

*TP, true positive; FP, false positive; FN, false negative; TN, true negative; PPV, positive predictive value; NPV, negative predictive value*.

## Discussion

In this study, ROP was detected in 21 and 63% of the patients with GA <32^+0^ and <28^+0^ weeks, respectively. The incidence of ROP was similar to that of a national survey (2010–2012) of 6,091 preterm infants from 22 hospitals in seven administrative regions in mainland China which showed that 25% (710/2861) and 67% (112/167) preterm infants with GA <32^+0^ and <28^+0^ weeks had ROP, respectively (3). Interestingly, we found a similar incidence of severe type I ROP in preterm infants with GA <32^+0^ and <28^+0^ weeks (22 and 20%, respectively). This is in contrast to the incidence observed in the mainland China and Shenzhen reports (8% and 12% at GA <32^+0^ weeks vs. 21% and 37% at GA <28^+0^ weeks; respectively) (3, 4). We believe that the different observations could be related to variation in screening and detection algorithms, and reporting mechanisms of various centers. Further, the management of very preterm neonates may also differ between centers depending on whether or not they practice “compassionate care” for critically ill neonates of GA >28 weeks based on non-medical reasons. However, the incidence of severe type I ROP in this study (4.7%) was high when we compared with that in a recent report of two French cohorts in 2008–2013 (0.8 and 2.3% of 3,669 and 1,272 very preterm infants) (18).

Retinopathy of prematurity is a multifactorial disease. Most studies have demonstrated that prematurity and low BW are the strongest predictive factors of ROP (19–21). Other factors associated with ROP include prolonged mechanical ventilation (22–24), the need for nasal CPAP (25, 26), development of BPD (27, 28), sepsis (29), hyperoxia (30), hypoxia (31), hypercapnia, and acidosis. Higher rates of ROP were found in infants with longer length of initial hospital stay (32, 33), and prolonged parenteral nutrition, which are a proxy for cumulative illness burden. We have similar findings in the current study.

The major finding in this study was that lower mean BP in the first 12 h after birth and other risk factors including those previously reported were significantly associated with development of ROP in univariate analyses (Table 1). While low BP could be a marker of disease acuity, hypotension and fluctuation of oxygen saturation might affect retinal perfusion and lead to retinal ischemia (34). Both mean BP and GA were predictors of ROP. However, the predictive accuracy of mean BP (AUC of 0.78) was not better than that of GA alone (AUC of 0.89), and the addition of mean BP did not improve ROP prediction further (AUC of 0.90). Nevertheless, this may suggest that mean BP was not a confounder here but was probably an effect modifier. Gestational age displayed sensitivity of 0.91 and specificity of 0.85 at the optimal cutoff value of 28.3 weeks for predicting the development of ROP, with positive and negative predictive values of 0.62 and 0.97, respectively. Mean BP in 0–12 h displayed sensitivity of 0.61 and specificity of 0.81 to predict ROP at the optimal cutoff value of 35 mmHg, and positive and negative predictive values were 0.47 and 0.88, respectively. When combining the variables of mean BP (0–12 h) and GA, sensitivity and specificity were 0.57 and 0.94, with positive and negative predictive values of 0.72 and 0.89, respectively. Regarding the accuracy rate, it was 0.86 of GA, 0.77 of mean BP (0–12 h), and 0.86 GA and mean BP (0–12 h) (Table 3). Interestingly, our data suggests that mean BP in the early hours after birth may have a modest effect and add positive predictive value to GA as perinatal risk factors for ROP. Studies using multiple cohorts of large sample size are required to examine the predictive value of BP in the early hours after birth.

There are several limitations of this study. First, this is a retrospective study of a small cohort of patients in a single unit which precluded us to examine effects of confounding variables in details and variables at birth including complications of delivery, apnea at birth, and resuscitation interventions (e.g., oxygen supplementation, oxygen saturation, and respiratory support). We also could not identify risk factors of severe ROP. Second, there was a wide confidence interval for odds ratio of BPD and sepsis in the prediction model, which might be in part attributed to a low incidence related to the small sample size. Third, we did not have digital fundus retinal photography for screening, which can improve objectivity and comparability of retinal findings. Last but not least, for total days of blood gas abnormalities during the hospital stay, the duration was the best estimate because we did not check blood gases daily.

In conclusion, in this small cohort of very and extremely preterm Chinese neonates, those survivors with ROP had lower blood pressure in the early hours after birth, in addition to younger gestation, longer hospital stay, and duration of acidosis, when compared to survivors without ROP. The high incidence of infants with ROP in our study emphasized the need for aggressive measures for prevention and control of the disease. We did not investigate but do believe that the safe implementation of oxygen and blood pressure therapy with appropriate monitoring, better antenatal and neonatal care, meticulous attention to hygienic procedures, and control of sepsis may reduce the incidence of ROP. Therefore, monitoring standards of neonatal care and conducting quality improvement projects across the country are essential for improving neonatal outcomes.

## Data Availability Statement

The raw data supporting the conclusions of this article will be made available by the authors, without undue reservation.

## Ethics Statement

The studies involving human participants were reviewed and approved by University of Hong Kong-Shenzhen Hospital Institutional Ethics Committee. Written informed consent from the participants' legal guardian/next of kin was not required to participate in this study in accordance with the national legislation and the institutional requirements.

## Author Contributions

H-BH designed the study, collected, analyzed and interpreted data, and drafted the initial manuscript. Y-HC designed the data collection instruments, collected data, and carried out the initial analyses. JW performed eye examinations, ROP diagnosis, clinical type, and treatment. MH, Q-SZ, Y-ZY, and C-BC conceptualized and designed the study, coordinated and supervised data collection, and critically reviewed important intellectual content. P-YC conceptualized and designed the study, designed the data collection instruments, interpreted the data, and critically reviewed important intellectual content. H-BH, Y-HC, JW, MH, Q-SZ, Y-ZY, C-BC, and P-YC reviewed, revised, and approved the manuscript. All authors contributed to the article and approved the submitted version.

## Conflict of Interest

The authors declare that the research was conducted in the absence of any commercial or financial relationships that could be construed as a potential conflict of interest.
